# Consumer providers' experiences of recovery and concerns as members of a psychiatric multidisciplinary outreach team: A qualitative descriptive study from the Japan Outreach Model Project 2011-2014

**DOI:** 10.1371/journal.pone.0173330

**Published:** 2017-03-03

**Authors:** Yoshifumi Kido, Mami Kayama

**Affiliations:** 1 Department of Psychiatric Nursing, Mie Prefectural College of Nursing, Tsu-city, Mie, Japan; 2 Department of Psychiatric and Mental Health Nursing, Graduate School of Nursing, St. Luke’s International University, Chuo-ku, Tokyo, Japan; University of Ottawa, CANADA

## Abstract

**Objective:**

The objective of this study was to clarify consumer providers (CPs) subjective experiences as members of a psychiatric multidisciplinary outreach team that provided services to individuals with a mental illness living in the community.

**Methods:**

A qualitative descriptive study was conducted through semi-structured interviews. Participants were clients hired as CPs in the Japanese Outreach Model Project from September 2011 until March 2014. Of the seventeen CPs, nine participated in this study. We looked at the CPs' subjective experiences of fulfillment and difficulty.

**Results:**

In the process of providing services, CPs experienced both achievements and concerns. They had a sense of achievement by caring for their clients and they experienced that they themselves were recovering. They were also concerned about having inadequate knowledge and skills to provide psychiatric services to their clients. Further, there were concerns about their dual role on the multidisciplinary team and being support staff while they were still using mental health services themselves.

**Conclusion:**

The results show that the activities of CPs included fulfillment, recovery, and dilemmas. Clarifications will likely contribute to an increase in understanding and cooperation between CPs and other professionals with whom they work. Further studies are needed to investigate policies related to mental health consumers who are also providers of mental health services.

## Introduction

In recent years the Japanese government has promoted policies to support people dealing with mental illness who continue to live in their communities. Additionally, programs have been implemented to address a variety of issues and to avoid hospitalization [1, 2]. The government offers support to allow such persons to continue living their lives in the community [3]. As part of this support, The Japanese Outreach Model Project (JOMP) was implemented from October 2011 until March 2014. In this project, community support was provided for both those with untreated mental illnesses, but who, because of their behavior, had received requests of assistance from health care centers. The project also included those who had suspended their treatment. For purposes of this report these two groups are referred to as clients. This support consisted primarily of visits by a psychiatric multidisciplinary team of specialists, resulting in controlling hospitalization or re-hospitalization [4]. The multidisciplinary outreach team consisted of psychiatrists, nurses, psychiatric social workers and occupational therapists. Additionally, as of 2013, the team composition also included clinical psychologists, pharmacists, and nutritionists (professions not included in the payment system for medical services), as well as mental health clients who were using psychiatric services that were brought on as ‘consumer providers’ (CPs).

CPs were defined in this study as “individuals with personal experience of serious mental illness who provide support services to others with serious mental illness, typically as clinical team members” [5]. CPs excelled at offering emotional support to clients who hesitate to participate in psychiatric treatment, and they were able to build relationships with those with whom it was difficult to create relationships by other methods. This was possible because consumer providers shared similar experiences with clients who were difficult to reach and had themselves changed their own behavior and improved their own skills [6]. Prior studies conducted in Western countries have demonstrated that services provided by multidisciplinary outreach teams who had CPs as team members were able to accomplish a variety of improvements in client outcomes. These included: an ability to quickly create treatment relationships, improve the quality of services, shorten periods of hospitalization, reduce re-hospitalization, lengthen the time spent living in the community, promote the recovery of the users of these services, and improve quality of life [7–11]. It has also been demonstrated that having CPs on multidisciplinary teams promoted the recovery of CPs. There was also a ripple effect of improving social awareness in the community as a whole and a reduction of the stigma associated with mental disorders among other professional team members who worked with CPs [12–14]. However, some studies demonstrated no significant results in terms of service outcomes and number of hospital days [15, 16] and the difficulties faced by CPs themselves were also apparent in some situations [14, 17, 18].

Some studies demonstrated that there were role conflicts and confusion, lack of policies and practices around confidentiality, poorly defined job structures and lack of support for peer supporters [19, 20]. As such, further studies are needed in order to examine various effects on clients as well as other professional members in multidisciplinary teams and in terms of the peer supporters themselves.

In recent years, the opportunities for CPs involvement have continued to grow in Japan. For example, CPs are providing support for people who are transitioning back into the community from long-term stays in psychiatric hospitals. This support comes in the form of helping clients adjust in their relationships with their families and by providing emotional support. However, there are no precedents in Japan of CPs offering community services as members of a multidisciplinary team in the form of visits to clients (individuals hesitant to participate in treatment and those who had suspended treatment). As such, further clarity is needed regarding support by CPs for clients who hesitate to participate in psychiatric treatment and CP subjective experiences as members of a multidisciplinary psychiatric outreach team. Given that the CPs also are concurrently managing their own mental illness, it is ethically imperative that the involvement of CPs in such services with the multidisciplinary team ‘do no harm’ and hopefully have a salutogenic effect.

### Study objective

The objective of this study was twofold. The first was to clarify how CPs felt about their subjective experiences as members of a psychiatric multidisciplinary outreach team when providing psychiatric services to untreated individuals and individuals who had suspended treatment. The second was to obtain suggestions about support policies for CPs and education about the CPs for other professionals working with them in psychiatric multidisciplinary outreach teams.

## Materials and methods

### Study design

This study is a qualitative descriptive study. Data were provided from semi-structured interviews using an interview guide. This was the first time in Japan for CPs to work with a multi-professional team. This was also the first time that other professionals had worked together with CPs. Thus, the purpose of this study was only to describe their subjective experiences. Since qualitative descriptive studies focus on discovering the nature of specific events, a qualitative descriptive methodology was most suitable for our purposes.

### Participants

The participants in this research were people who worked as CPs in the Japanese Outreach Model Project [4] from September 2011 until March 2014. Their team psychiatrists or directors recruited CPs to the JOMP. This meant that CPs had dual roles as a peer supporter and also as client. They were officially employed by each organization through the project budget. There were 37 teams/organizations that provided multidisciplinary psychiatric outreach and a total of 17 CPs who were members of the multidisciplinary outreach team. We invited all 17 CPs working in this model project to participate in our study and nine agreed.

In this project, the CPs were not specially trained as a consumer providers because certified training programs were not available at that time in Japan. However, most of the CPs in this study were WRAP [21] facilitators prior to this model project, and in workshops they learned and gained an understanding about “What is (personal) recovery” [22–24]

The majority of CPs were males in their 40s diagnosed with schizophrenia, schizotypal, delusional, or other non-mood psychotic disorders (coded ICD-10 F2) (see Table 1). The average amount of prior experience as a CP was 34 months. There were two participants who were taking CPs developmental training during the time the interviews were conducted. At the time of the interview the average number of clients visited at home was eight and average length of time participating in JOMP was 17 months. (Table 1)

**Table 1:** Characteristics of study participants.

	Age range	Diagnosis (ICD-10)	Previous Experience as a Consumer-provider	Months participating in outreach service	Number of home visits
A	30s	F2	Yes	15	1
B	40s	F2	Yes	23	4
C	40s	F2	Yes	4	0
D	30s	F2	No	16	7
E	40s	F3	No	17	10
F	40s	F2	No	17	10
G	40s	F2	Yes	17	4
H	40s	F3	No	22	0
I	60s	F2	Yes	23	2

Note: F2 = Schizophrenia, schizotypal and delusional disorders; F3 = Mood (affective) Disorders

### Data collection

The data were collected between April 2013 and March 2014. The interviews were conducted in locations chosen by the participants and where they felt their privacy was protected. Moreover, it was explained to the participants that the interviews would range from 30 to 60 minutes so that it would not be a burden on them. The actual time of the interviews ranged from 25 to 64 minutes with an average time of 40 minutes.

The researchers conducted the semi-structured interviews using an interview guide. Interview guides contained how CPs felt in terms of subjective fulfillment and difficult experiences encountered when providing psychiatric services to untreated individuals and individuals who had suspended treatment as members of a psychiatric multidisciplinary outreach team. The interview guide was developed in consultation and cooperation between the authors and research collaborators who were experienced with semi-structured interviews. During the interview, facial expressions and speech and participant conduct was also observed and documented. The interviews were recorded upon gaining consent from the participants. The recorded media was rendered anonymous.

### Analytical method

This study looked at the CPs' subjective experiences as members of a psychiatric multidisciplinary outreach team with a focus on their sense of fulfillment and difficulties. Data analysis was conducted using a qualitative descriptive method [25, 26]. After carefully reading the transcripts of the digitally recorded interviews, we extracted several themes from CPs’ experiences, along with detailed content. At this stage of the analysis, we did not attempt conceptualization of the extracted data; rather, we maintained the contexts and actual words used by the participants. The process of CPs experiences was listed according to the timeline of the situation. Then we named each step of their experience using their *in-vivo* words. We (authors) carefully discussed whether the names accurately reflected the experiences we observed in the data and arrived at a consensus.

### Ethical considerations

This study was conducted after receiving approval from St. Luke's College of Nursing Research Ethics Committee (approval number: 13–006).

Potential participants were given a written and oral explanation of the following points. The study objective, data and methods would not be used for anything other than the study purpose, the data would provide anonymity, be coded, and would be stored under lock and key. After the study was concluded, the data would promptly be discarded. It was also explained that if a person did not consent to the study, it was not to their disadvantage, and that if any of the participants requested that their participation be withdrawn, they were provided with a form to withdraw their consent to the study. Additionally, at this time the content of the interview that had been given up to that point would be discarded. The participants and the people in charge of the organizations that they belonged to then signed research consent forms to give their consent.

It was also explained in an appropriate manner that the interview could be stopped at any time or that the interviewee might stop the interview. Thus, considerations were made to ensure that there would be no psychological detriment to study participants.

## Results

The results that emerged from the analyses of the CPs’ interviews were seven sub-categories that conceptually supported two categories (Table 2). In the following explanations of the categories and subcategories, the narratives of the study participants were put in "quotation marks."

**Table 2:** The subjective experiences of the CPs as members of the multidisciplinary outreach team.

Category	Sub-category
1. Recovery became possible through support	1) Earning trust through shared experiences
	2) Receiving feedback from recovering clients
	3) Using own experience within the multidisciplinary team
	4) Gaining self-confidence while continuing to work and managing own health
2. Concerns about consumer providers’ expertise and their dual roles	1) Worrying about having inadequate knowledge and skills to provide psychiatric services to clients
	2) Feeling unsure about how to support clients whose illness and or experience differed from their own
	3) Bothered by their complicated role as concurrent service user and provider

### 1. Recovery became possible through support

#### 1) Earning trust through shared experiences

All CPs recalled having a period where they felt resistance to accepting their mental illness and receiving services. This was much the same experience as their clients had. During this period they did not want to acknowledge that they had an illness, wanted to hide their illness, and did not want to go to the hospital. During this difficult time, the CPs and clients they were supporting felt isolated because even if they were asking for help, they could not find someone with whom to consult. Thus, they both experienced a difficult situation where they needed to continue their lives in their communities while in a state of feeling the ‘difficulty of living’.

“I want to make the most of my difficult experiences with my illness to help the people I'm supporting to be connected with medical exams and to become better”. “I want to make the most of my experiences with my illness, and after becoming sick, I had several years of difficult thoughts, so I strongly felt that I since I went through these hard thoughts I wanted them to somehow be useful somewhere”.

When the clients were directly faced with this type of situation and expressed sentiments like, ‘I want someone to understand this feeling’, ‘I want to talk to someone who understands’, or ‘I don't know what I should do’, the CPs felt like this was the perfect time for them to talk about their own experiences. From their difficult experiences the CPs knew that when supporters would talk about things, it was not pleasant and that there were things you could talk about to a supporter. The CPs felt like they were able to earn trust by having these shared experiences with the people they were supporting.

“I think back on the things I've experienced one more time… I remember the times that were hard for me, and… I think of what kind of state this person is in and feel like I should talk to them about my own experiences of this type”. “When messages like, ‘doesn't anyone understand this feeling’ or ‘you guys don't understand, who cares about this feeling, or I've become sick are stated, I say ‘no, I understand’”.

#### 2) Able to get feedback from the recovering clients

By talking about their own experiences the CPs were able to create a relationship of mutual trust among the multidisciplinary team and the client. This allowed treatment and support to be provided more smoothly. The CPs were able to feel a sense of achievement and the feeling that their actions were worthwhile by seeing the gradual recovery of clients for whom they provided support. In particular, as the interaction started to engage clients and when they became more conversant, CPs remarked,

“I felt really good about sympathizing with the person. I told them about my own experiences and about seeing the person receiving support gradually getting better. When their facial expressions changed and became bright I felt really good”.

Moreover, clients who had recovered gave positive feedback to the CPs who had provided them with support. The CPs then felt a sense of happiness about their clients and verbalized proactive attitudes and feelings and going forward with life.

“When I hear words like ‘It's good that I'm alive, right’ or ‘I've had a lot of hard times, and it's hard right now, but I've become able to think about my own life going forward’ or ‘As I've talked about my own difficulties, it has become easier’, I feel happy”.

#### 3) Having one's own experiences while within the multidisciplinary team

The experience of working together and sharing support methods and the goal of supporting untreated individuals and individuals who had suspended treatment with regional community officials and an outreach team comprised of many professionals from the psychiatric social work field was a first, even for some people with CPs experience. The perspective and profession of the supporter made a difference as to how the client was approached. As they touched on the distinctive characteristics of various expertise and skills, the CPs realized that there was some knowledge and viewpoints that only they possessed. They felt that they were able to fully use this on the team and in the community.

“When someone said ‘the situation that this person is in right now felt like this, so because my circumstances were similar, so isn't that why I know the types of things they are thinking about?’ I was able to reply. That made me feel a slight sense of achievement. I'm not sure if it is a sense of achievement, rather I think I felt positive about myself or like I had done something”.

Furthermore, the CPs were able to get support because they could consult with other professions when they were having a difficult time dealing with their clients. The environment of respecting each other's specializations truly allowed the CPs to feel that their unique expertise was fully utilized.

“I think that because there are professionals who are there, they are to some extent supporting me too. So when we do group work I think my experiences are being fully utilized, so it isn't just me who is projecting my experiences because I'm in a team where they are fully utilized. I don't think I could do this type of thing alone”.

#### 4) Being able to continue working while managing health provided self-confidence

The CPs remained users of psychiatric services. They continued their outreach work while always feeling the anxiety of working while maintaining their own mental health. In reality the CPs had days where they faced their worries completely on their own and could not sleep; yet, the feeling that their work as CPs was useful to those they were supporting gave them courage. This, together with the stable income as compensation for their work, was tied to their sense of self-confidence and feelings of self-affirmation.

“Once my physical condition and health were better, I initially had anxieties about being able to properly work throughout a full year”. “The systematic life of repetition, of waking up every day at a designated time and going to work gradually gave me self-confidence, and I think this and earning money was the best”.

### 2. Concerns about consumer providers’ expertise and their dual roles

#### 1) Worrying about having inadequate knowledge and skills to provide psychiatric services to clients

While the CPs felt that their own viewpoints and experiences of symptoms, treatment, and recovery were fully utilized on the multidisciplinary team, they also worried that they had inadequate fundamental or specialized psychiatric knowledge and skills, which other specialists had. Psychiatrists and nurses had knowledge and skills about medications, side effects, and somatic care. Similarly, public health nurses and psychiatric social workers had knowledge about social and governmental support.

These worries came from being in contact with other professionals' skills, expertise and approaches to dealing with clients. The CPs were concerned that, aside from their own experiences, they lacked sufficient skills and the expertise necessary to give support.

“I don't have any expertise like a nurse, psychiatric social worker or psychologist because I haven't ever studied those things”. “I wondered if I could get along with a multidisciplinary team. I realized that I didn’t have enough psychiatric knowledge to support clients when I discussed situations with other specialists in the team. I have been worrying about this”.

Additionally, they were worried about whether their own actions were really helpful or not. Subsequently, these pressures also caused them to worry.

“I keep thinking that ‘I have to work hard and be highly evaluated by other staff because I have been employed’. ‘I have to do something useful for clients’. ‘I have to attain some progress immediately’”.

#### 2) Feeling unsure about how to support clients except for the illness that was personally experienced

Each CP had dealt with individual and different experiences in their symptoms, treatment and medications, social support and services, family support or conflicts, and recovery processes, even if they were diagnosed with the same illness. Thus the CPs shared what they had actually experienced with clients who were dealing with the same or similar experiences or illness. However, in the JOMP, the CPs sometimes supported people who were dealing with experiences or illnesses that the CPs had not experienced. In these cases, the CPs were concerned that they did not know how to reach their client.

“I have thought that I can't say whether or not this is really good for this person because I haven't been in a similar situation”.

Additionally, in the multidisciplinary conferences that considered treatment and care plans of clients with other professionals, the CPs had their own particular thoughts and opinions, but they were worried that their own thoughts might not be correct because they did not have similar experiences and this became a source of anxiety for them.

“I can really only talk about the things that have happened in my own experiences, so I am very concerned that because I don't have that type of expertise and so I might not be really useful”.

#### 3) Bothered by the their complicated position and role of concurrently being a user and supporter

The CPs were bothered about the complicated position of being both a user of psychiatric services as well as a service provider. This could be seen in several situations that arose as a member of the multidisciplinary team and service provider.

“I got used to working with team members. This might mean that I started to be conscious of my standpoint as a member of a multidisciplinary team”.

One situation that arose was that the CPs were unsure of how to connect with friends with whom they had been in day-care or in other communities prior to this point. The CPs were anxious about the possibility of supporting their friends as a service provider. And if that was the case, it bothered them as to what to talk about and what to do for their friends.

“I'm a patient, and until very recently I've also been a day-care user, and I'm also staff. This is a three-fold relationship”. “Even if I've socialized with other patients, isn't this the first time I've socialized with them as an outreach member? That… um. That position is a bit different, so I'm wondering what will happen with that part of it”.

Another situation they were concerned about was returning to their status as ‘just a patient’ and their relationship with the other members of their multidisciplinary team after the model project was completed.

“Don’t the nurses and medical personnel come in to work at the same place for a long period of time? I've been coming into work for this project, but once this project is done and its time is up, I will just be another patient. I find myself thinking ‘What will people think of me?’”

## Discussion

The CPs worked as members of a psychiatric multidisciplinary team to provide outreach for diagnosed, but untreated individuals, and individuals who had suspended their treatment. Their experience was wide-ranging. In the process they felt worried and anxious about their complex position in a dual role on the multidisciplinary outreach team as both a patient themselves and as support staff, and sometimes even in three roles. Nevertheless, they found a sense of achievement through the recovery of the people they were supporting, and they felt that they were also recovering themselves.

### 1. A feeling of achievement from supporting participant recovery was also tied to the recovery of the consumer providers

Recovery is defined as a process in which people are able to live, work, learn, and participate fully in their communities; it involves the development of new meaning and purpose in one’s life despite a disability or mental disorder [21]. This was a central concept of the psychiatric social work in Europe and America during the 1980s and 90s, which has in recent years been permeating into Japan [22]. This concept can be organized into eight components: “being supported by other people, re-establishing hope and determination, re-defining the self and accepting the illness, participation in meaningful activities and expanding social roles, managing symptoms, and regaining control and responsibility" [23].

The CPs in this study were able to receive support that fully utilized the characteristics of the multidisciplinary team. The CPs were supported by having the team as partners who could support and advise them when they were worried about how to handle or deal with their clients. The CPs also had the team as support who had a good understanding when the CPs were troubled about managing their own physical condition. Moreover, the CPs were able to make the fullest use of their unique viewpoints and characteristics within the multidisciplinary team. This resulted in positive feedback for the support they provided to clients. This feedback also came in the form of seeing the recovery of their clients. Thus, they were able to participate in socially meaningful activities and feel fulfillment from their own roles. The self-confidence they gained by continuing to be able to work while managing their own physical and mental health despite their illness allowed them to be able to regain self-control and responsibility. In short, the CPs progressed in their own recovery process by supporting the recovery of other clients in need of support, to the extent it became a transformative experience.

The positive influence of the CPs own actions on their own recovery has been shown in prior studies in both Western countries and also in Japan [27, 28]. This research found similar results. CPs activities within the multidisciplinary outreach team had a positive impact on the recovery of the CPs.

### 2. Worrying about being in a complicated position of concurrently being a service user and member of the support staff

The CPs continued their actions as members of a multidisciplinary team that offered support while continuing to be service users themselves. During this time, they worried about their relationships with their friends with whom they had socialized as fellow service users. The idea of fellow members being on equal footing with the CPs during self-help and peer support activities is one that is carefully protected. Nevertheless, there were concerns that CPs' taking actions as supporters might violate this idea. Prior research in Europe and America has shown that CPs offering support to their own friends and trying to continue a friendship led to CPs bewilderment about the unclear boundary lines [17, 29]. The results of this research seem to show that CPs in Japan face similar dilemmas.

Another anxiety that was faced by the CPs arose because there were many professional staff on the multidisciplinary team who were known to the CPs from their day-care or outpatient programs or who were their attending physicians. Because of this, the CPs were concerned about what the staff thought about them. Another uncertainty that the CPs had was if they would return to being in the relationship of service users with staff once their activities on the multidisciplinary team were completed. There have been reports in Western countries of CPs having a service provider and service user relationship with people on the team prior to becoming team members. In such cases, problems arose around creating relationships where although CPs were on equal footing as professionals with other team members they continued to be thought of as service users [17, 30–32]. In addition to the aforementioned issues with friendships between CPs and clients, there were also similar dilemmas that arose in the relationships between the CPs and other professionals on the team. This seemed to be another source of anxiety for the CPs.

One solution that was suggested for the dilemma of being in this kind of complex situation involved hiring CPs from other organizations where they had not established a relationship of being service users with the team [15, 29]. It seems that definite considerations are essential when creating future multidisciplinary teams that include CPs and peer support activities. The importance of maintaining an appropriate boundary and the conflict that can arise from the CPs new role are clearly identified in Japan's "*Second Edition of the Guidelines for Training Peer Support Specialists for People with Mental Disorders*" that was based on the America's National Association of Peer Specialists' Guidelines. However, further research is needed on specific policies to solve the dilemmas that are characteristic of a multidisciplinary team.

### 3. To fully demonstrate the specialized role of consumer providers on the multidisciplinary team

The CPs felt the necessity of having expertise and skills outside of their own experiences through working together with other professionals in a variety of settings. The CPs were also worried about what were their specializations and unique points. Among the CP participants in this research there were CPs who had experiences with peer support when in need of consultation and when being discharged from a hospital. This project was started at the same time that the aforementioned developmental training began for "Peer Specialists for People with Mental Illness." Therefore, it had not yet spread nationwide, and the CPs began working with clients before they had the opportunity to receive proper systematic training for their new roles. This lack of training seemed to be one of the causes of CPs anxiety.

Moreover, prior research has indicated that role and relationship confusion arose between CPs and other professionals on their teams. This had, at times, caused disorder on a team [15, 29]. The results of this research did not reveal any confusion of this type between the CPs and other professionals. However, because this is an analysis of interviews conducted in the final year of the project, and it is possible that the results reflect systems and relationships of mutual assistance that had already been built through trial and error. Thus, it may have been that differences from the different professions and roles had been overcome. However, it is possible that when the team was initially formed it faced difficulties similar to those identified by prior research. Therefore, it seems to be essential to clarify how a good cooperative system can be cultivated while concurrently investigating a training system that provides the necessary expertise for a project, creates internal support within the team, and also cultivates external support.

### 4. Limitations

This study has several limitations. Firstly, it was possible for participants to define the word “recovery” depending on their own knowledge and understanding. Until recently, in Japan the term “recovery” [21–23] would only indicate “clinical recovery,” (“*kaifuku*” in Japanese) where the target was to improve upon symptoms. It was possible that for many professionals, including CPs and clients, their use and understanding of the word “recovery” also included “clinical recovery,” as the terms are somewhat unclear in Japan. However, most CPs in this study learned and understood the phrase, “What is (personal) recovery?” because most of them are WRAP [24] facilitators and learned about this at workshops before this model project. Secondly, the results obtained in this study were a product of an analysis of interviews with CPs about their experiences in the model project that was conducted as a pilot initiative. The model project, however, did not have developmental training for CPs that was specifically for CPs working on an outreach team with untreated individuals and individuals who had suspended treatment. Thus, it is possible that the content that was expressed in the interviews was impacted by whether or not the CPs had previous experience working on projects of this nature. Moreover, it is possible that the results were influenced by differences in the member composition of each team that the CPs belonged to and the differences in team management systems. Additionally, the results may have been impacted by differences in actual services provided during visits and the number of clients who were offered support. Thirdly, the interviewer of this study was one of coordinators of this project and a nurse, which may have affected the openness of the participants. Fourthly, this study did not ask about the attitudes of other professionals of the team and stigma experiences. Further studies are needed with such a focus.

Despite the above research limitations, this study provided further clarification about recovery and feelings of anxiety that CPs experienced as members of a psychiatric multidisciplinary outreach team. The findings here will likely contribute to increasing understanding and cooperation between CPs and nurses, psychiatrists, psychiatric social workers, occupational therapists and other professionals with whom they work.
